# Supplementary material for: Decoding a cryptic mechanism of metronidazole resistance among globally disseminated fluoroquinolone-resistant Clostridioides difficile
Source: Nat Commun. 2023 Jul 12;14:4130. doi: 10.1038/s41467-023-39429-x (PMC10338468; doi:10.1038/s41467-023-39429-x)
Supplement: Supplementary file 1 — Supplementary Information [file 41467_2023_39429_MOESM1_ESM.pdf]

Supplementary Information

Decoding a cryptic mechanism of metronidazole resistance among globally disseminated fluoroquinolone-resistant *Clostridioides difficile*

Abiola O. Olaitan[1,2*], Chetna Dureja[1*], Madison A. Youngblom[3], Madeline A. Topf[3], Wan-Jou Shen[1], Anne J. Gonzales-Luna[4], Aditi Deshpande[1], Kirk E. Hevener[5], Jane Freeman[6,7], Mark H. Wilcox[6,7], Kelli L. Palmer[8], Kevin W. Garey[4], Caitlin S. Pepperell[9,10#], Julian G. Hurdle[1#]

[1]Center for Infectious and Inflammatory Diseases, Institute of Biosciences and Technology, Texas A&M Health Science Center, Houston, Texas, USA

[2]Department of Biology, University of Waterloo, Waterloo, Ontario, Canada

[3]Microbiology Doctoral Training Program, University of Wisconsin-Madison, Madison, Wisconsin, USA

[4]Department of Pharmacy Practice and Translational Research, University of Houston College of Pharmacy, Houston, Texas, USA

[5]Department of Pharmaceutical Sciences, College of Pharmacy, University of Tennessee Health Science Center, Memphis, Tennessee, USA

[6]Department of Microbiology, Leeds Teaching Hospitals Trust, Leeds, UK

[7]Healthcare Associated Infection Research Group, School of Medicine, University of Leeds, Leeds, UK

[8]Department of Biological Sciences, University of Texas at Dallas, Richardson, Texas, USA

[9]Department of Medical Microbiology and Immunology, School of Medicine and Public Health, University of Wisconsin-Madison, Madison, Wisconsin, USA

[10]Department of Medicine, Division of Infectious Diseases, University of Wisconsin-Madison, Madison, Wisconsin, USA

* These authors contributed equally

[#] These authors jointly supervised this work

Corresponding to: cspepper@medicine.wisc.edu; jhurdle@tamu.edu

Table of contents of supplementary information

**Supplementary Figure 1**	Relative frequency distributions of metronidazole MICs against 405 *C. difficile* strains
**Supplementary Figure 2**	The effect of heme on the transcriptome of epidemic R20291 in response to metronidazole toxicity, and comparison to non-epidemic CD196
**Supplementary Figure 3**	Comparison of growth of isogenic strains
**Supplementary Figure 4**	Identification and validation of *nimB* as a mechanism of heme-dependent resistance to metronidazole
**Supplementary Figure 5**	Overlay of *Bacteroides thetaiotaomicron* NimB crystal structure
**Supplementary Figure 6**	Effect of heme binding His-55 site in *Cd*NimB on heme-dependent metronidazole resistance
**Supplementary Figure 7**	Visual and spectroscopic analysis of *Cd*NimB indicates it is a heme-binding flavin enzyme
**Supplementary Figure 8**	Assay development and analysis of nitroreductase activity of *Cd*NimB
**Supplementary Figure 9**	Comparison of cellular reduction of 2-nitroimidazole to 2-aminoimidazole
**Supplementary Figure 10**	Heme-dependent metronidazole resistance is exhibited by *Bacteroides fragilis* NimA
**Supplementary Figure 11**	FST outlier analysis identifies association between *nimB* promoter and *gyrA* mutations with HMR
**Supplementary Figure 12**	Transcriptional analysis of *nimB* in two heme-dependent metronidazole-resistant strains

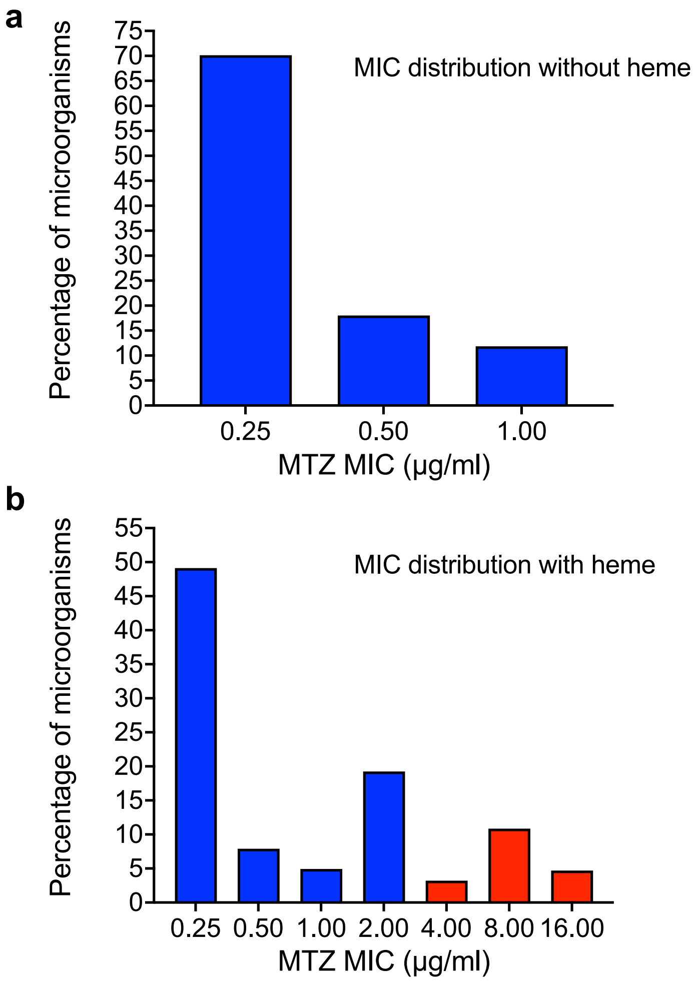

Supplementary Figure 1. **Relative frequency distributions of metronidazole MICs against 405 *C. difficile* strains.** Susceptibility testing done either in the absence (**a**) or presence (**b**) of heme showed heme increased the frequency of strains exhibiting resistance (red bars); this is based on the EUCAST epidemiological cutoff (breakpoint) of >2 µg/ml for metronidazole-resistant strains. The panel includes strains with MICs of 0.25 µg/ml and 1 µg/ml, without and with heme respectively, and are considered to display heme-dependent resistance to metronidazole.

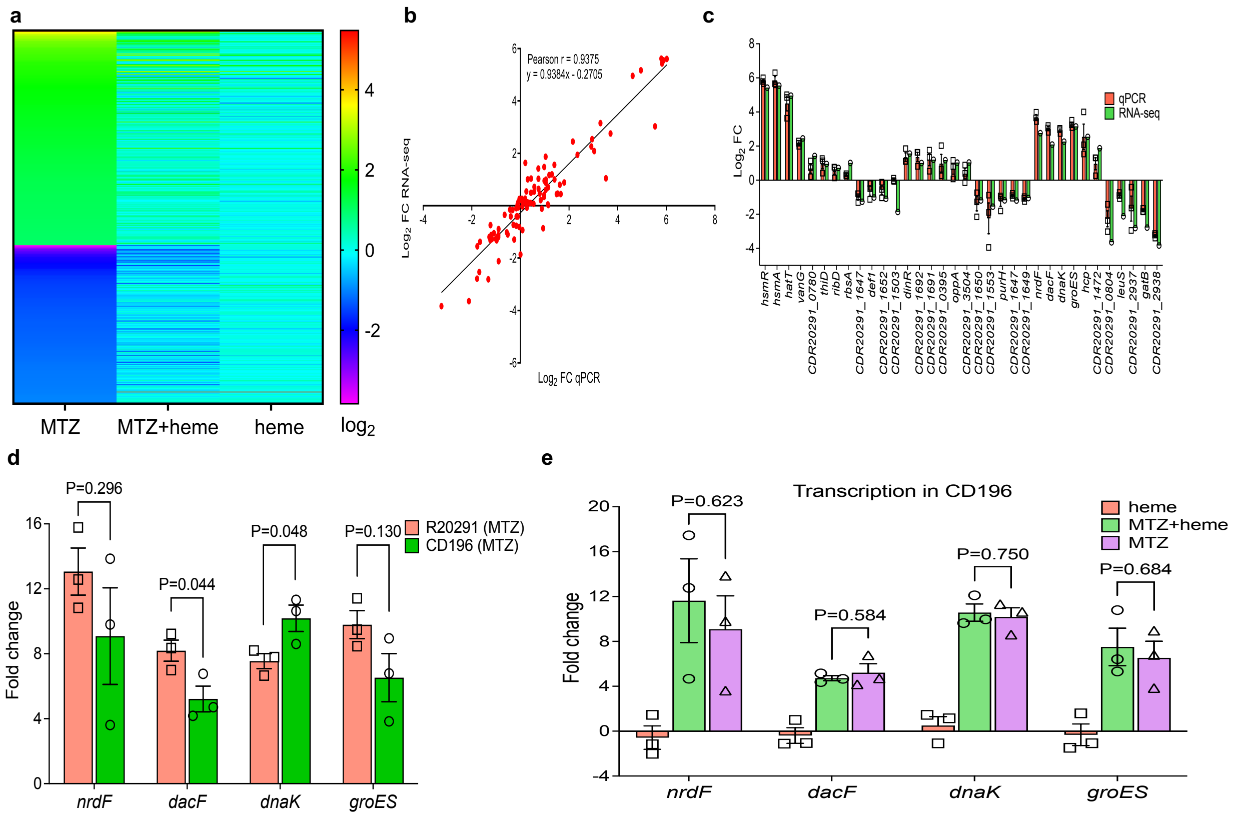

Supplementary Figure 2. **The effect of heme on the transcriptome of epidemic R20291 in response to metronidazole (MTZ) toxicity, and comparison to non-epidemic CD196. (a)** Heat map showing pattern of differentially expressed genes (DEG) in R20291 in MTZ (2 μg/ml), MTZ plus heme (5 μg/ml) or heme alone. DEGs were determined using an FDR cutoff of ≤ 0.01 and an absolute value of Log_2 Fold Change ≥ 2, relative to DMSO exposed culture. **(b)** Pearson correlation plot from a panel of 34 genes (20 upregulated and 11 downregulated). Data analyzed by simple linear regression in GraphPad Prism 9.4.1 indicated a correlation coefficient (R^2) of 0.9384 from equation $y = 0.9384*x – 0.2705$ with statistical significance of F-value 725.4 and P<0.0001. Of the three biological replicates from RNA-seq, one was discarded due to poor quality in FASTQC, but gene expression was validated by qPCR using three biological replicates. **(c)** Transcription of selected genes from the above RNA-seq and qPCR data; data represent the means ± standard error of mean of three biological replicates for qPCR data and two biological replicates for RNA-seq data. **(d)** In the absence of heme, CD196 and R20291 display a similar transcription pattern. **€** In MTZ-susceptible CD196, heme does not attenuate MTZ responsive gene transcription, unlike in R20291 (see Fig. 1d of the main text). Data in d and e are plotted as the means ± standard error of mean from three biological replicates; statistical significance of data in d and e were assessed by two-tailed multiple unpaired t test with correction for multiple comparisons using the Holm-Šídák method and alpha set to 0.05.

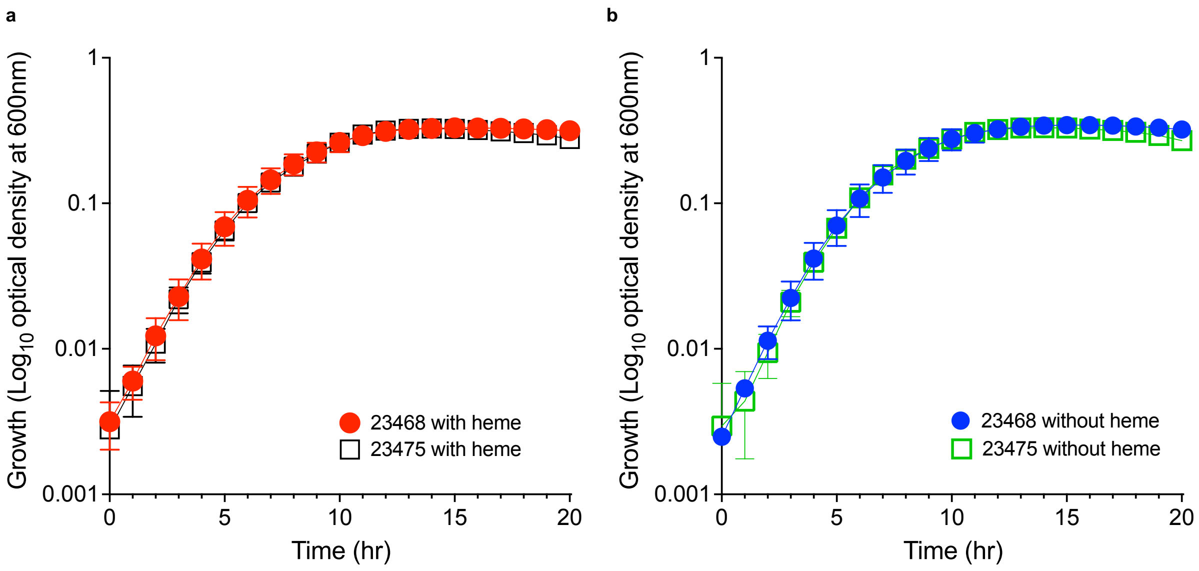

Supplementary Figure 3. Comparison of growth of isogenic strains. 23475 (susceptible) and 23468 (resistant) show comparable growth curves in BHI broths, with heme (**a**) or without heme (**b**); shown are the means ± standard error from three biological replicates.

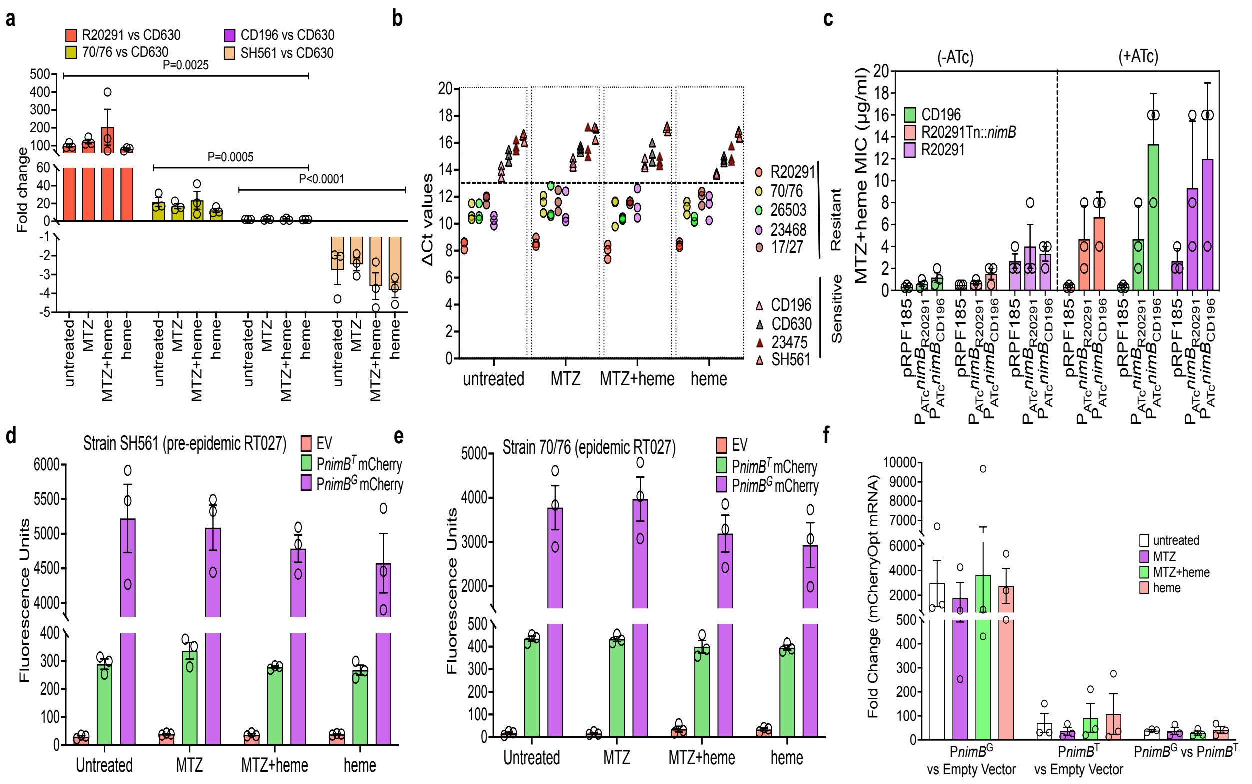

Supplementary Figure 4. **Identification and validation of *nimB* as a mechanism of heme-dependent resistance to metronidazole (MTZ). (a)** qPCR analysis of *nimB* transcription in resistant and susceptible strains with respect to the susceptible lab strain CD630, under different conditions. Data represent the means ± standard error of mean of three biological replicates; statistical significance was assessed for all compiled data on each strain by Welch and Brown-Forsythe one-way ANOVA with Dunnett's Multiple Comparison in GraphPad Prism 9.4.1. **(b)** Relative cycle threshold (Ct) values of *nimB* transcripts among resistant (R20291, 70/76, 23468, 25603, 17/27) and susceptible (CD196, CD630, SH561, 23475) isolates under different conditions. Ct values were normalized to the housekeeping 16S rRNA. Data represent three biological replicates and each value is shown in the aligned dot plot. **(c)** MICs of MTZ upon overexpression of CD196 and R20291 *nimB* ORFs under the control of the ATc-inducible promoter (P*tet*). Expression was induced by 64 ng/ml ATc; data represent the means ± standard error of mean of three biological replicates. **(d, e)** Further comparison of promoter strengths of P*nimB*^{G} and P*nimB*^{T} based on mCherryOpt transcription in SH561 and 70/76, susceptible and resistant, respectively. Fluorescence was normalized to OD_{600}nm culture density; MTZ MICs in heme were 0.25 µg/ml and 8 µg/ml against SH561 and 70/76, respectively. The fluorescence of P*nimB*^{G} was higher across all test conditions, indicating constitutive expression, supporting findings in Fig. 2f of the main text. **(f)** mCherry mRNA levels were also higher with P*nimB*^{G} than *Pnim*^{T}; expressions were performed in CD196. Data in d to f represent the means ± standard error of mean of three biological replicates.

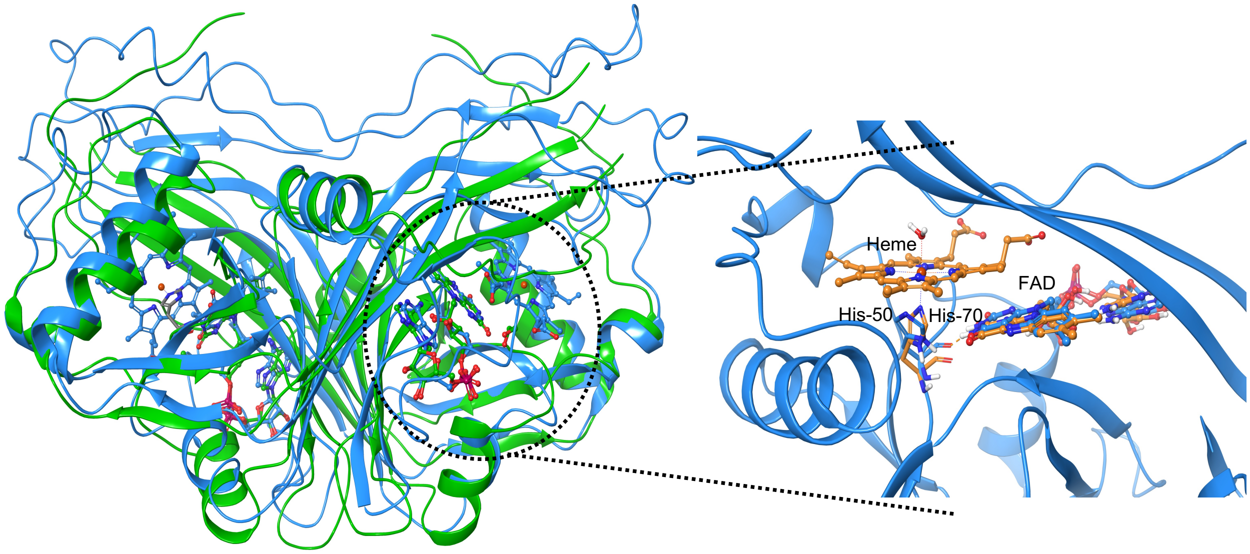

Supplementary Figure 5. Overlay of *Bacteroides thetaiotaomicron* NimB crystal structure (PDB 2FG9, blue ribbons, resolution 2.20 angstrom) with Anf3 (PDB 6RK0, green ribbons, resolution 0.99 angstrom). Both previously published proteins are dimers and closely align; the close-up shows histidine-50 in *Bt*NimB is homologous to the heme binding proximal ligand histidine-70 of Anf3. *Bt*NimB was co-crystalized with FAD, while Anf3 was co-crystalized with both heme and FAD; the FAD cofactors of both proteins occupy the same relative positions in their cognate proteins. The *Bt*NimB structure was used to generate a homology model of *Cd*NimB in Figure 3b of the main text. *Bt*NimB (2FG9 [https://www.rcsb.org/structure/2FG9]) Anf3 (6RK0 [https://www.rcsb.org/structure/6RK0]).

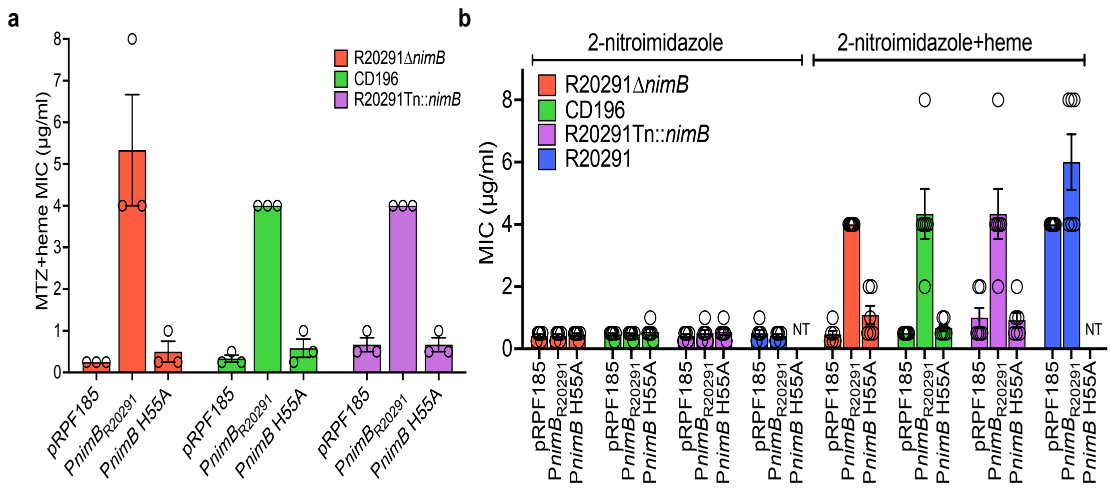

Supplementary Figure 6. Effect of heme binding His-55 site in *Cd*NimB on heme-dependent metronidazole (MTZ) resistance. (a) The His55Ala (i.e., H55A) mutation does not confer resistance, or wildtype level resistance, when expressed in various MTZ-susceptible strains; wildtype or H55A variant was expressed in plasmid pRPF185 under P*nimB*^{G} promoter in the indicated strains (MTZ-susceptible CD196, R20291-*Tn::nimB* and R20291Δ*nimB*). MICs are shown as means ± standard error of mean from three biological replicates, done in heme containing agars. **(b)** Analysis of MICs of 2-nitroimidazole in the absence and presence of heme against abovementioned MTZ-susceptible strains, with MTZ-resistant R20291 as a control. MICs are shown as means ± standard error of mean from six biological replicates.

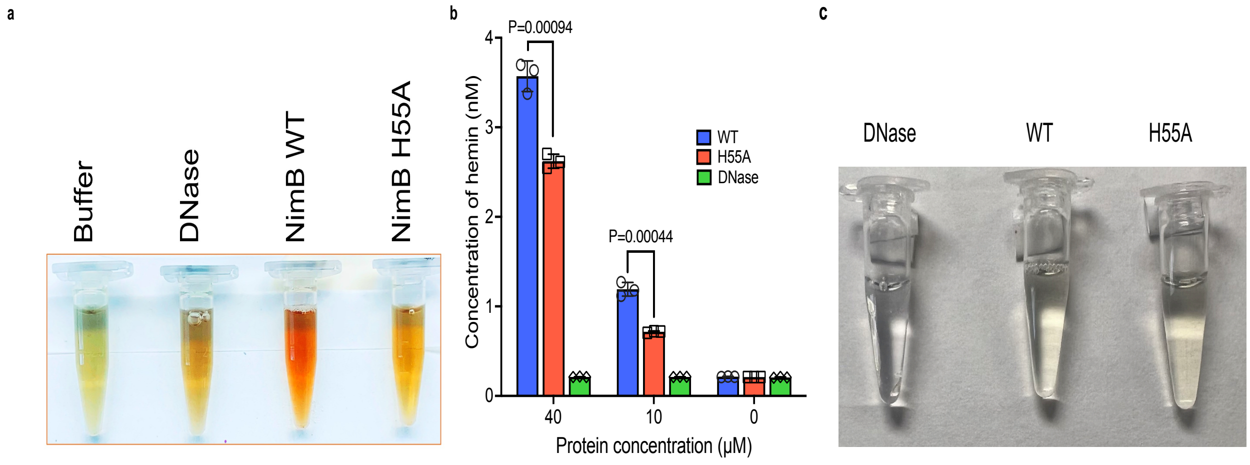

Supplementary Figure 7. Visual and spectroscopic analysis of *Cd*NimB indicates it is a heme-binding flavin enzyme. (a) Upon addition of heme to wildtype *Cd*NimB, a characteristic reddish-brown color is observed, which indicates heme-protein complexation. In contrast, with heme, the H55A variant has a yellow orange color, similar to that of the non-heme-binding DNase control. Each tube contains pooled samples from heme binding experiments. **(b)** Semi-quantitative detection of intracellular heme bound by wildtype *Cd*NimB and its H55A variant. Following expression and purification in *E. coli* BL21(DE3), the relative content of heme was measured in a peroxidase colorimetric assay to detect low concentrations of heme, against a hemin standard. Peroxidase uses heme as a cofactor in the reaction. While the results show the WT and mutant *Cd*NimB proteins contain heme, these values are semi-quantitative, and based on heme from *Cd*NimB's becoming available to the peroxidase. Plotted are the means ± standard error of mean from three experimental replicates and results of wildtype and H55A *Cd*NimB compared; data was statistically analyzed in Graphpad prism 9.4.1 by two-tailed multiple unpaired t test with correction for multiple comparisons using the Holm-Šídák method and alpha set to 0.05. **(c)** Purified *Cd*NimB has a light-yellow color, indicative of the presence of flavin(s), which is consistent with Nim being flavin proteins.

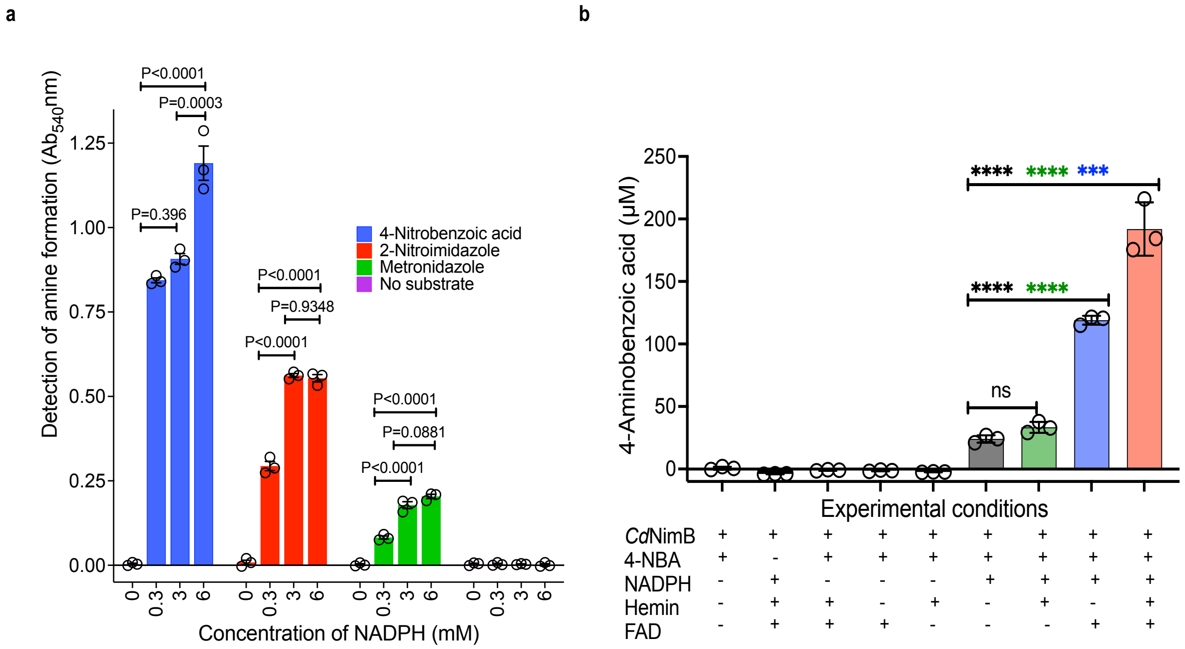

Supplementary Figure 8. Assay development and analysis of nitroreductase activity of *Cd*NimB. (a) *Cd*NimB nitroreductase activity was dependent on NADPH concentration and presence of a nitroaromatic substrate. The assay contained wildtype *Cd*NimB (10 µM), heme (10 µM), FAD (10 µM), NADPH (0.3, 3 or 6 mM) and a nitroaromatic substrate (5 mM). Reactions were incubated for 2 h, and the formation of aromatic amines detected using the Bratton-Marshall assay. There was no significant difference in production of 4-aminobenzoic acid from 4-nitrobenzoic acid when 0.3 to 3 mM of NADPH was used, but production increased with 6 mM of the substrate; for 2-nitroimidazole and metronidazole, the maximum production of their amino derivatives appeared to plateau at 3 mM of NADPH. **(b)** Nitroreductase activity was assessed with or without 3 mM of NADPH, FAD (10 µM), heme (10 µM), *Cd*NimB enzyme, and/or 4-nitrobenzoic acid (5 mM). Results showed *Cd*NimB needs NADPH and a nitro-substrate; addition of both FAD and heme produced the highest activity; activity in the absence of FAD or heme is likely due to *Cd*NimB having natively bound FAD and heme during protein expression in *E. coli* (see Supplementary Fig. 7). Data in a and b are from three experimental replicates; plots show the mean ± standard error of mean; and their statistical significance were assessed by one-way ANOVA with Tukey's Multiple Comparison in GraphPad Prism 9.4.1; ***P=0.0002, ****P<0.0001 and ns= not significant (P=0.7414).

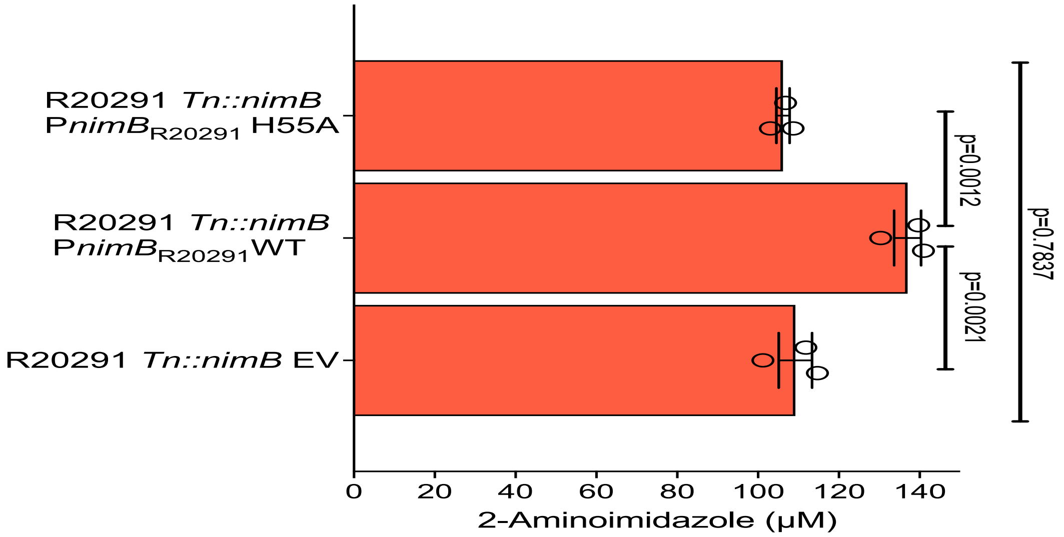

Supplementary Figure 9. Comparison of cellular reduction of 2-nitroimidazole to 2-aminoimidazole by R20291-*Tn::nimB* complemented with either wildtype R20291 *nimB*, His55Ala codon mutant or the empty vector (EV, pRPF185). The strain complemented with wildtype *nimB* showed a 25.4-29.0% increase in 2-aminoimidazole production, compared to that complemented with the Ala-55 variant or the empty vector. Concentrated cultures were treated with 2-nitroimidazole (2 mM) for 3 h, before 2-aminoimidazole was quantified by LC-MS/MS at Texas A&M University (see methods). Data represent the means ± standard error of mean of three biological replicates; statistical significance was assessed by one-way ANOVA with Tukey's Multiple Comparisons in GraphPad Prism 9.4.1.

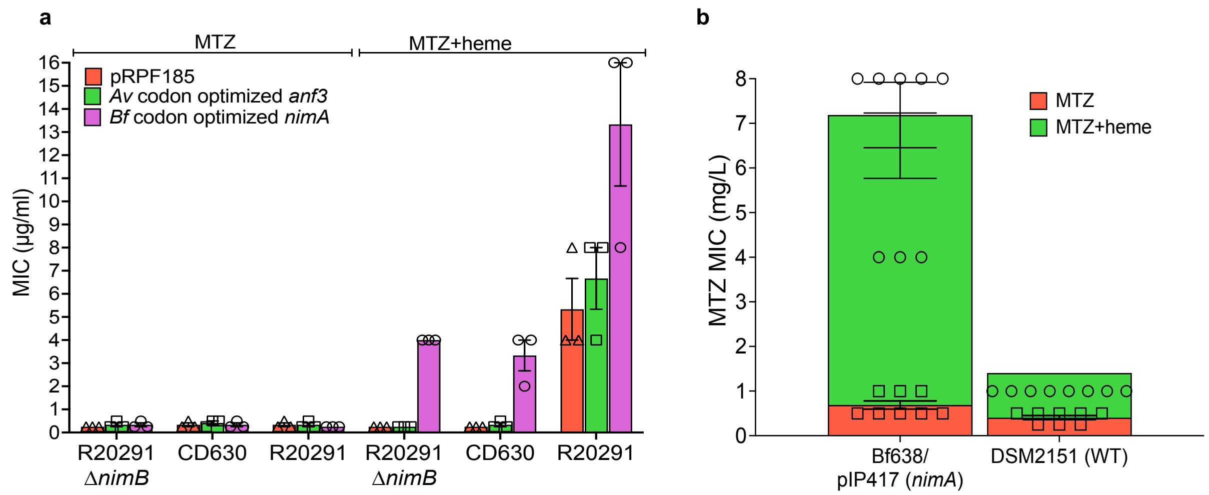

Supplementary Figure 10. Heme-dependent metronidazole (MTZ) resistance is exhibited by *Bacteroides fragilis* NimA. (a) Recombinant NimA from *B. fragilis* (*Bf*) confers heme-dependent MTZ resistance in susceptible strains and increases resistance in R20291. While Nim proteins (e.g., *Bf*NimA and *C. difficile* NimB are structurally related to Anf3, the latter did not confer heme-dependent MTZ resistance). Genes encoding *Bf* and *Av* proteins were codon-optimized for expression in *C. difficile*. **(b)** Susceptibility testing of *B. fragilis* known metronidazole-resistant strain Bf638/ pIP417 (*nimA*) and susceptible in the presence and absence of heme. Bf638/ pIP417 (*nimA*) showed that its resistance to metronidazole is heme-dependent; Bf638/ pIP417 (*nimA*) encodes a plasmid-borne *nimA*. DSM2151is a *B. fragilis* susceptibility testing control strain that is susceptible to metronidazole. Data in a represent the means ± standard error of mean of three biological replicates; data in b is from of four biological replicates, with each having a technical replicate.

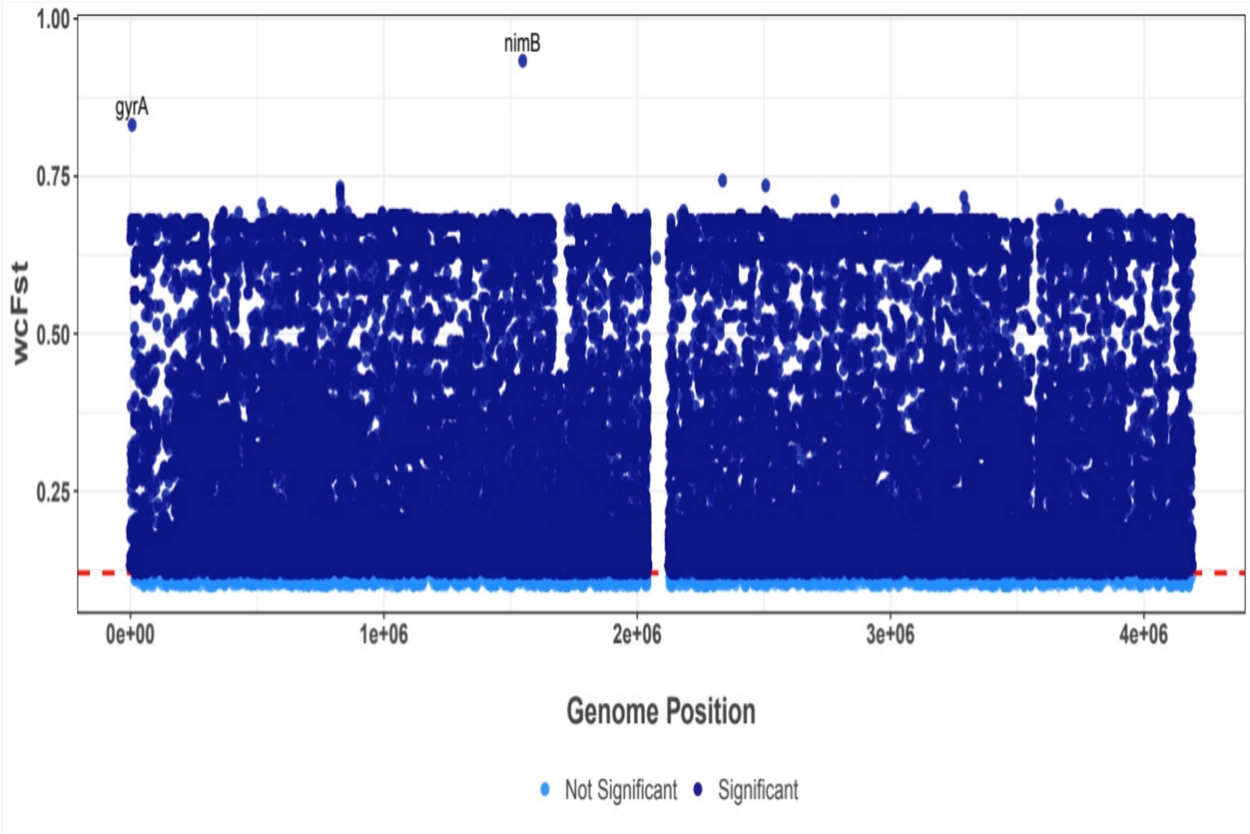

Supplementary Figure 11. FST outlier analysis identifies association between *nimB* promoter and *gyrA* mutations with HMR. Weir & Cockerham's Fixation index (FST) was calculated for all SNPs, comparing heme-dependent metronidazole-resistant (HMR) and susceptible (S) populations. FST values are plotted by genome position. Both *gyrA* and *nimB* SNPs showed exceptionally high FST values, indicating extreme frequency differences between HMR and S populations.

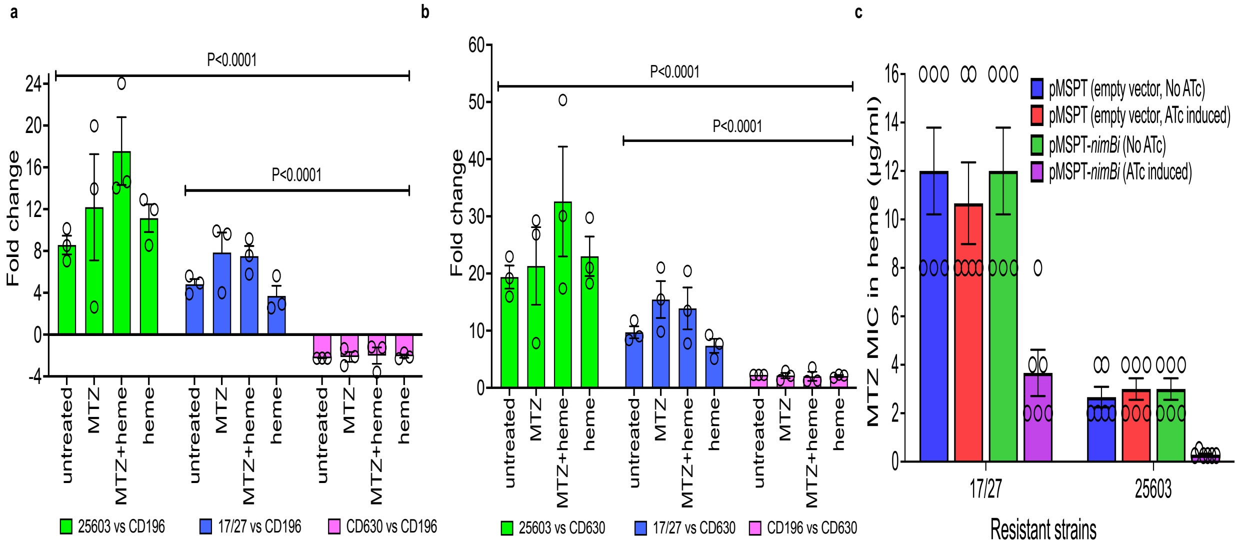

Supplementary Figure 12. Transcriptional analysis of *nimB* in two heme-dependent metronidazole-resistant strains. (a, b) qPCR analysis of *nimB* transcription in strains 17/27 and 25603 with respect to the susceptible strains CD196 or the lab strain CD630, under different conditions. Data represent the means ± standard error of mean of three biological replicates; statistical significance was assessed for all compiled data on each strain by Welch and Brown-Forsythe one-way ANOVA with Dunnett's Multiple Comparison in GraphPad Prism 9.4.1. **(c)** Genetic silencing of *nimB* reduced MTZ resistance in 17/27 or completely reverses it in 25603. *nimB* was silenced by an antisense RNA (asRNA), which was induced by anhydrotetracycline (64 ng/ml) from P*tet* promoter in vector pMSPT. MICs are shown as means ± standard error of mean from six biological replicates.
