# Supplementary material for: Decoding a cryptic mechanism of metronidazole resistance among globally disseminated fluoroquinolone-resistant Clostridioides difficile
Source: Nat Commun. 2023 Jul 12;14:4130. doi: 10.1038/s41467-023-39429-x (PMC10338468; doi:10.1038/s41467-023-39429-x)
Supplement: Supplementary file 3 — Description of Additional Supplementary Files [file 41467_2023_39429_MOESM3_ESM.pdf]

Description of Additional Supplementary Files:

Supplementary Data 1. Strains of C. difficile studied.

Supplementary Data 2. Primers used in this study.

Supplementary Data 3. Genetically constructed strains from this study.
