# Supplementary material for: Decoding a cryptic mechanism of metronidazole resistance among globally disseminated fluoroquinolone-resistant Clostridioides difficile
Source: Nat Commun. 2023 Jul 12;14:4130. doi: 10.1038/s41467-023-39429-x (PMC10338468; doi:10.1038/s41467-023-39429-x)
Supplement: Supplementary file 5 — Reporting Summary [file 41467_2023_39429_MOESM5_ESM.pdf]

nature portfolio

Corresponding author(s): Caitlin Pepperell, Julian Hurdle

Last updated by author(s): May 18, 2023

Reporting Summary

Nature Portfolio wishes to improve the reproducibility of the work that we publish. This form provides structure for consistency and transparency in reporting. For further information on Nature Portfolio policies, see our Editorial Policies and the Editorial Policy Checklist.

Statistics

For all statistical analyses, confirm that the following items are present in the figure legend, table legend, main text, or Methods section.

n/a	Confirmed	
☐	☒	The exact sample size (*n*) for each experimental group/condition, given as a discrete number and unit of measurement
☐	☒	A statement on whether measurements were taken from distinct samples or whether the same sample was measured repeatedly
☐	☒	The statistical test(s) used AND whether they are one- or two-sided<br>*Only common tests should be described solely by name; describe more complex techniques in the Methods section.*
☒	☐	A description of all covariates tested
☐	☒	A description of any assumptions or corrections, such as tests of normality and adjustment for multiple comparisons
☐	☒	A full description of the statistical parameters including central tendency (e.g. means) or other basic estimates (e.g. regression coefficient) AND variation (e.g. standard deviation) or associated estimates of uncertainty (e.g. confidence intervals)
☐	☒	For null hypothesis testing, the test statistic (e.g. *F*, *t*, *r*) with confidence intervals, effect sizes, degrees of freedom and *P* value noted<br>*Give P values as exact values whenever suitable.*
☒	☐	For Bayesian analysis, information on the choice of priors and Markov chain Monte Carlo settings
☒	☐	For hierarchical and complex designs, identification of the appropriate level for tests and full reporting of outcomes
☐	☒	Estimates of effect sizes (e.g. Cohen's *d*, Pearson's *r*), indicating how they were calculated

Our web collection on statistics for biologists contains articles on many of the points above.

Software and code

Policy information about availability of computer code

Data collection	All applicable software and codes used are stated and cited in the Methods, and can be readily accessed through the below links, along with information to operate the tools. The following software were used: GraphPad prism 9.4.1; RBS Calculator v2.1; Phyre2; HemoQuest (http://131.220.139.55/SeqDHBM/); JCat platform (http://www.jcat.de); Prime software in 2020-2 Schrödinger molecular modelling suite; software on Galaxy platform (https://usegalaxy.org/) as follows TrimGalore (galaxy version 0.4.3.1), FastQC (galaxy version 0.72+galaxy1) and multiFastQC (galaxy version 1.7), htseq-count (galaxy version 0.6.1galaxy3), BWA-MEM program (galaxy version 0.7.17.1); edgeR on Degust platform (http://degust.erc.monash.edu/); bcl2fastq (v2.20.0.422); Reference-guided assembly pipeline (https://github.com/pepperell-lab/RGAPepPipe.git); Picard v1.183 (http://broadinstitute.github.io/picard/); TrimGalore v0.6.4 (https://github.com/FelixKrueger/TrimGalore); Fst outlier analysis using vcflib (https://github.com/vcflib/vcflib) and bespoke scripts (https://github.com/myoungblom/Evolution_of_the_GGI/tree/main/Figure5); pySEER analysis is available at https://github.com/myoungblom/cdiff_gwas.
Data analysis	See above.

For manuscripts utilizing custom algorithms or software that are central to the research but not yet described in published literature, software must be made available to editors and reviewers. We strongly encourage code deposition in a community repository (e.g. GitHub). See the Nature Portfolio guidelines for submitting code & software for further information.

Data

Policy information about availability of data

All manuscripts must include a data availability statement. This statement should provide the following information, where applicable:

- Accession codes, unique identifiers, or web links for publicly available datasets
- A description of any restrictions on data availability
- For clinical datasets or third party data, please ensure that the statement adheres to our policy

The raw whole-genome sequencing data generated in this study have been deposited in NCBI database under Bioproject numbers PRJNA914992 (https://www.ncbi.nlm.nih.gov/bioproject/PRJNA914992), PRJNA943263 (https://www.ncbi.nlm.nih.gov/bioproject/PRJNA943263), PRJNA555597 (https://www.ncbi.nlm.nih.gov/bioproject/PRJNA555597), PRJNA940988 (https://www.ncbi.nlm.nih.gov/bioproject/PRJNA940988); all accession numbers for individual strains are provided in Supplementary Data 1. Raw RNA-Seq data generated in this study were deposited in NCBI database under accession number PRJNA880780 (https://www.ncbi.nlm.nih.gov/bioproject/?term=PRJNA880780). Source data are provided with this paper. Raw data for histograms in various figures are found in the Source Data. Supplementary Data list primers used in this study. Supplementary Data 3 list genetically constructed strains from this study. pySEER analysis is available at https://github.com/myoungblom/cdiff_gwas. All data and materials in this study are available to any researcher for the purpose of reproducing or extending the study's results.

Research involving human participants, their data, or biological material

Policy information about studies with human participants or human data. See also policy information about sex, gender (identity/presentation), and sexual orientation and race, ethnicity and racism.

Reporting on sex and gender	Not applicable.
Reporting on race, ethnicity, or other socially relevant groupings	Not applicable.
Population characteristics	Not applicable.
Recruitment	Not applicable.
Ethics oversight	Not applicable.

Note that full information on the approval of the study protocol must also be provided in the manuscript.

Field-specific reporting

Please select the one below that is the best fit for your research. If you are not sure, read the appropriate sections before making your selection.

[x] Life sciences [ ] Behavioural & social sciences [ ] Ecological, evolutionary & environmental sciences

For a reference copy of the document with all sections, see nature.com/documents/nr-reporting-summary-flat.pdf

Life sciences study design

All studies must disclose on these points even when the disclosure is negative.

Sample size	Sample size calculations were not conducted; samples sizes were based on use of minimum number of samples required for biological and experimental significance to be established. Throughout a minimum of three independent samples were used throughout and defined in figure and table legends.
Data exclusions	Data excluded were initial workup experimental data for enzyme assays, to establish best conditions for the enzyme assays. Related workup conditions are described in the manuscript. We could not establish conditions for electron paramagnetic resonance (EPR) and this data was excluded; heme binding studies were done three times and all arose at the same conclusion, as in workup experiments, that heme binding is reduced in the mutant compared to the wildtype; reported are the data from optimized assays (done on three replicates and representative data is shown); data from unoptimized assays pointed to same conclusion. An RNAseq replicate was excluded becaused it failed FastQC, but the data for the remaining two replicates were validated in qRT-PCR. Inter-laboratory MICs were not included.
Replication	Biological experiments were replicated independently by the two first co-authors on different days and reviewed by co-author Hurdle; the indepdent experiments came to the same conclusion. Bioinformatics were conducted by different methods that came to the same conclusions and reviewed by Pepperell.
Randomization	Randomization was not performed in biological experiments as samples were treated to the same conditions without variation in inter-sample handling. Strains carrying different genetic traits were subjected to the same test conditions to determine influence of the genetic trait on phenotype. To identify wcFst outliers we repeated our analysis 100X using randomly assigned phenotypes and used the maximum wcFst value observed in this null distribution as a significance cut-off.
Blinding	Blinding of samples was not performed. Key to the experiments was susceptibility tests detecting resistance; the susceptibility tests protocol was therefore independently reviewed by authors Garey and Luna and the experimental protocol independently validated between the two

labs as being able to identify metronidazole resistance.

Reporting for specific materials, systems and methods

We require information from authors about some types of materials, experimental systems and methods used in many studies. Here, indicate whether each material, system or method listed is relevant to your study. If you are not sure if a list item applies to your research, read the appropriate section before selecting a response.

Materials & experimental systems

n/a	Involved in the study
☒	☐ Antibodies
☒	☐ Eukaryotic cell lines
☒	☐ Palaeontology and archaeology
☒	☐ Animals and other organisms
☒	☐ Clinical data
☒	☐ Dual use research of concern
☒	☐ Plants

Methods

n/a	Involved in the study
☒	☐ ChIP-seq
☒	☐ Flow cytometry
☒	☐ MRI-based neuroimaging
